# Portable Take-Home System Enables Proportional Control and High-Resolution Data Logging With a Multi-Degree-of-Freedom Bionic Arm

**DOI:** 10.3389/frobt.2020.559034

**Published:** 2020-09-25

**Authors:** Mark R. Brinton, Elliott Barcikowski, Tyler Davis, Michael Paskett, Jacob A. George, Gregory A. Clark

**Affiliations:** ^1^Biomedical Engineering, University of Utah, Salt Lake City, UT, United States; ^2^Ripple Neuro, Salt Lake City, UT, United States; ^3^Neurosurgery, University of Utah, Salt Lake City, UT, United States

**Keywords:** bionic arm, myoelectric prostheses, proportional control, Kalman filter, take-home

## Abstract

This paper describes a portable, prosthetic control system and the first at-home use of a multi-degree-of-freedom, proportionally controlled bionic arm. The system uses a modified Kalman filter to provide 6 degree-of-freedom, real-time, proportional control. We describe (a) how the system trains motor control algorithms for use with an advanced bionic arm, and (b) the system's ability to record an unprecedented and comprehensive dataset of EMG, hand positions and force sensor values. Intact participants and a transradial amputee used the system to perform activities-of-daily-living, including bi-manual tasks, in the lab and at home. This technology enables at-home dexterous bionic arm use, and provides a high-temporal resolution description of daily use—essential information to determine clinical relevance and improve future research for advanced bionic arms.

## Introduction

Electromyography (EMG) from the residual forearm has been used to control commercially available and research-grade prosthetic arms (Kuiken et al., 2016; Hargrove et al., 2017; Ottobock, 2017; Touch Bionics Inc, 2017; Wendelken et al., 2017; George et al., 2018; Page et al., 2018; Perry et al., 2018; Mobius Bionics, 2020). Although research has demonstrated proportional control of multiple, independent degrees of freedom (DOFs) (Davis et al., 2016; George et al., 2018; Page et al., 2018), commercially available prostheses still suffer from a variety of limitations (Biddiss and Chau, 2007), including limited number of pre-determined grips (Touch Bionics Inc, 2017), temporal delay due to sequential inputs used to select grips (Ottobock, 2017; Mobius Bionics, 2020), fixed output force (e.g., from traditional classifier algorithms) (Resnik et al., 2018a), extensive training that lasts days to weeks (Resnik et al., 2017, 2018a, 2019), and non-intuitive methods of control [e.g., inertial measurement units (IMUs) on residual limb or feet] (Resnik et al., 2018b; Mobius Bionics, 2020).

Dexterous control of multiple DOFs, and the training associated with them, are not always amenable to deployment on portable systems with limited computational power, and as a result only a few pattern-recognition (i.e., classifiers) (Kuiken et al., 2016; Resnik et al., 2017; Mastinu et al., 2018; Simon et al., 2019) or direct control algorithms have been studied at home (Pasquina et al., 2015; Simon et al., 2019). A Kalman filter (Wu et al., 2006), modified with non-linear, *ad-hoc* adjustments (George et al., 2019a; Nieveen et al., in review) can provide a computationally efficient approach (George et al., 2020c) to study proportionally and independently controlled multi-DOF prostheses at home. Proportional control algorithms enable realistic and life-like prosthetic control and can induce device embodiment in transradial amputees (Page et al., 2018).

High temporal resolution of the position and forces applied to the prosthesis is necessary to describe the interactive and refined movements made possible with proportionally controlled prostheses. These data are also necessary to describe key aspects of actual prosthesis use: revealing when objects were manipulated; whether movements were performed unilaterally or bilaterally (for bilateral amputees); which grasps were preferred; how often each DOF was used; and when new inter-digit collaborative movements were employed.

Preliminary at-home use of this portable, prosthetic control system, capable of providing six-DOF, real-time, proportional control was published previously (George et al., 2019a). Here we describe the portable system and the tasks completed at home in greater detail, including how the modified Kalman filter is trained and implemented on the portable system, as well as the system's ability to record an unprecedented dataset of EMG, hand positions, and force sensor values. This technology constitutes an important step toward the commercialization of dexterous bionic arms by demonstrating at-home use and the ability to record prosthesis use with high temporal resolution.

## Materials and Methods

### Design Considerations

A portable take-home system designed to research advanced bionic arms should meet several criteria for optimal performance and data collection: (a) the system must accurately and efficiently control the prosthesis; (b) training of the control algorithm must not be too long or burdensome to prevent its daily use—and thus should include the ability to quickly load a previously trained control algorithm; (c) high-temporal-resolution data should be stored automatically so that researchers can study at-home use without influencing the users with in-person observation; and (d) the system must be easy to use and allow the user to adjust control preferences.

#### Accurate and Efficient Control

For accurate and efficient control, the system must be able to record EMG from the residual forearm, predict new kinematic positions, and send those positions to the prosthesis quickly with minimal or no perceived delay between the intention to move and the movement itself. Previous work in our lab has demonstrated responsive control of prostheses at update cycles of 33 ms (30 Hz) using a modified Kalman filter (Wendelken et al., 2017; George et al., 2018; Page et al., 2018; Kluger et al., 2019). The goal of this work was to implement these algorithms on a portable computer and provide position updates with minimal delay between the user intent (muscle activation) and the prosthesis movement. We have shown that updates at 33 ms provide responsive control and lead to embodiment of the physical prosthesis (Page et al., 2018). Updates at this speed are also within the optimal controller delay for prosthesis control (Farrell and Weir, 2007).

#### Fast Training for Daily Use

For daily use, training of the control algorithm should be intuitive and fast. The time required to train a control algorithm includes data collection while the participant mimics preprogrammed movements of the prosthesis (George et al., 2020d), and training of the control algorithm itself (e.g., training the modified Kalman filter matrices). When training, or retraining, is required, it should be as fast as possible to minimize the setup time prior to use. Lengthy setup and training could make advanced prostheses burdensome to incorporate into daily life and prevent their acceptance among amputees. The system should also allow reloading of a previously trained control algorithm on demand.

#### Comprehensive Record of Unsupervised Arm Use

A common approach to measure prosthesis use is to place IMUs on the prosthesis and record movement acceleration and angular velocity (Hargrove et al., 2017; Resnik et al., 2017, 2018b; Graczyk et al., 2018). However, this approach fails to discriminate between gross movements from the residual limb and actual movement of the prosthesis's hand and wrist. Video collection via body cameras can be used to record actual prosthesis use and other metrics (such as compensation strategies), but require storage of large video files and time-intensive *post-hoc* analyses (Spiers et al., 2017). Furthermore, the presence of a video camera reminds study participants they are being watched even though lab personnel are not physically present. However, with a portable system, prosthesis use at home can be studied by recording every movement for each DOF. By also recording the force applied to DOFs, interactive prosthesis use can be discerned from passive arm movements, such as those that might occur during walking or exploratory hand movements that are not functionally directed. Beyond describing total prosthesis use, this rich dataset can reveal detailed, refined movements and collaborative interactions between DOFs—including the force applied with each movement.

#### User-Friendly Control Adjustments

Finally, a prosthetic control system should be easy to use and allow adjustments to fit unique preferences. This includes a quick and simple approach to turn the system on, train the control algorithm and load a previously trained control algorithm. Control adjustments could also include flexibility to lock a DOF during dexterous tasks to prevent unwanted movements—for example locking the thumb and solely using the index finger could provide a more stable pinch. In addition, feedback from participants in our lab suggest the system should also provide users flexibility to operate specific DOFs (e.g., wrist) in a velocity-control mode (Kluger, 2019).

### Hardware and Signal Acquisition

**Figure 1:**
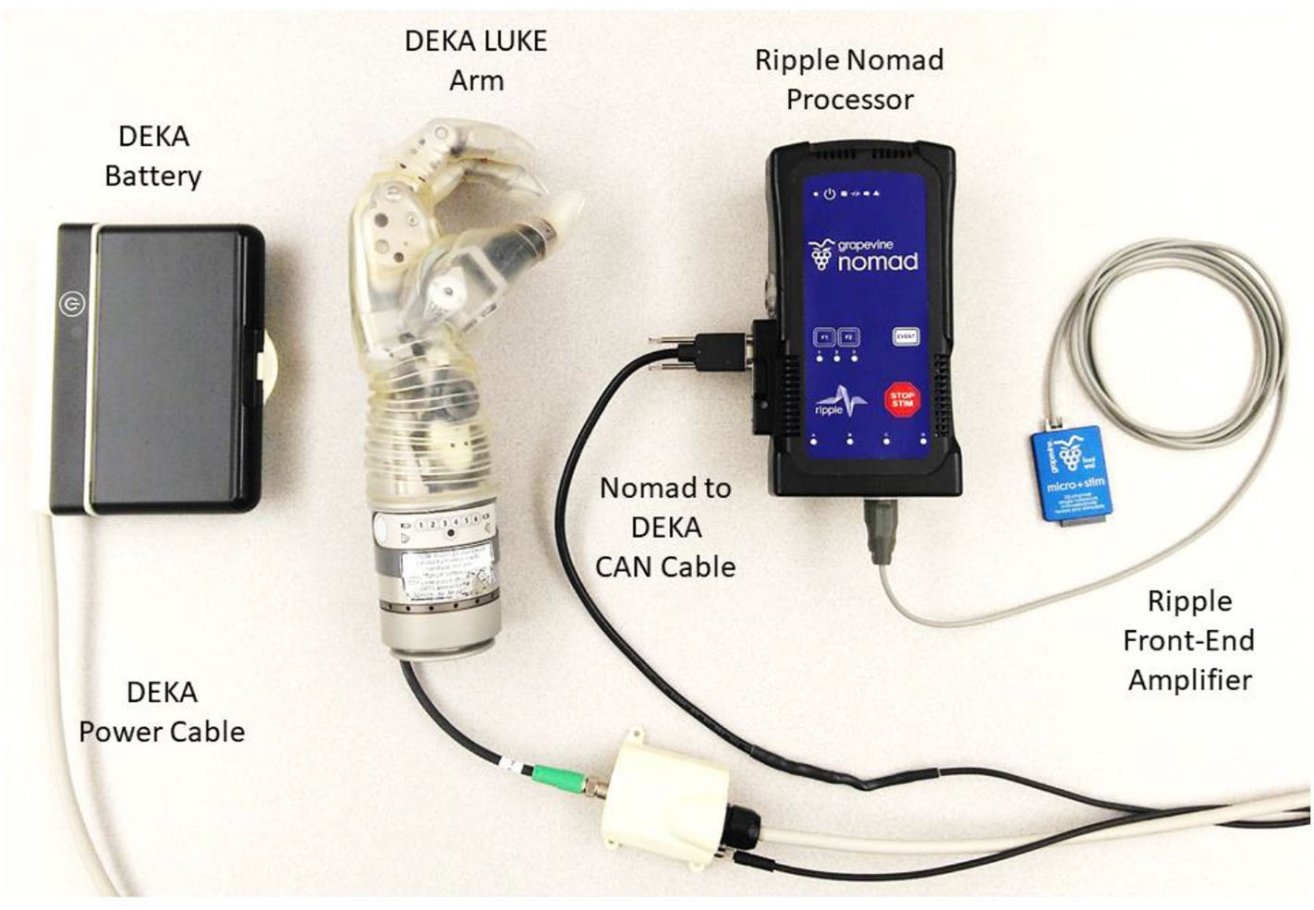
Portable take-home system for dexterous prosthetic control. The Ripple front-end acquires, filters, and amplifies EMG (at 1 kHz) to estimate motor intent using a modified Kalman filter with the battery powered Nomad neural interface processor. Communication occurs using a CAN protocol with the DEKA LUKE Arm to send commanded movements to the arm (at 30 Hz) and receive back the actual kinematic positions for six DOFs and the forces from 13 sensors (nine torque sensors, four pressure sensors at 30 Hz).

The components of the portable system are shown in Figure 1. The DEKA LUKE Arm (DEKA; Manchester NH, USA) has 6 DOFs including thumb (D1) adduction/abduction; D1 flexion/extension; index (D2) flexion/extension; coupled middle, ring, and pinky (D3–D5) flexion/extension; wrist flexion/extension, which also includes a slight radial and ulnar deviation, respectively; and wrist pronation/supination. It also has 19 sensors: six that report the position of each DOF and 13 that report the forces on each digit—including four directions on D1, two on the D2, and one on each of D3, D4, and D5—and on the lateral, dorsal, and palmar (distal and proximal) aspects of the hand. The prosthesis itself records the aggregated use (i.e., time) within bins of movement velocity and electrical current draw for each DOF. It also records the total time each sensor experienced various forces (ten bins from zero to a max of 25.5 N) and the total time each DOF spent in various positions (ten bins across range of motion, which varies by DOF). We designed a custom python socket so that our compiled algorithms could communicate with and store data from the DEKA LUKE Arm's CAN-BUS interface (at 30 Hz).

For the prosthetic control algorithm and data storage we used the Nomad neural interface processor (Ripple Neuro; Salt Lake City, UT, USA) for several reasons: an external, exchangeable battery provides up to 4 h of power; wireless communication to external devices; 500 GB of hard disk storage; and up to 512 channels for data acquisition and stimulation. We modified the Ripple firmware provided with the Nomad so that our compiled control algorithms could directly: acquire, filter and store EMG (1 kHz); start and stop via external buttons; and communicate over WiFi with external devices (TCP socket). Using a front-end amplifier (Figure 1; Ripple Neuro, Salt Lake City, UT, USA) we filtered (15 to 375 Hz bandpass; 60/120/180 Hz notch) the implanted EMG (iEMG) or surface EMG (sEMG, both were sampled at 1 kHz). sEMG in intact participants was recorded with a Micro + Stim front-end (Ripple Neuro, Salt Lake City, UT, USA), and iEMG in the amputee participant was recorded with an active gator front end (Ripple Neuro, Salt Lake City, UT, USA). The Nomad runs Linux 8 (jessie) environment with an Intel^®^ Celeron™ processor (CPU N2930) at 1.83 GHz with 2-GB RAM. Control algorithms were converted to C using MATLAB^®^ Coder and compiled for stand-alone use on the portable Nomad.

### EMG Feature Calculation and Decoding of Motor Intent

**Figure 2:**
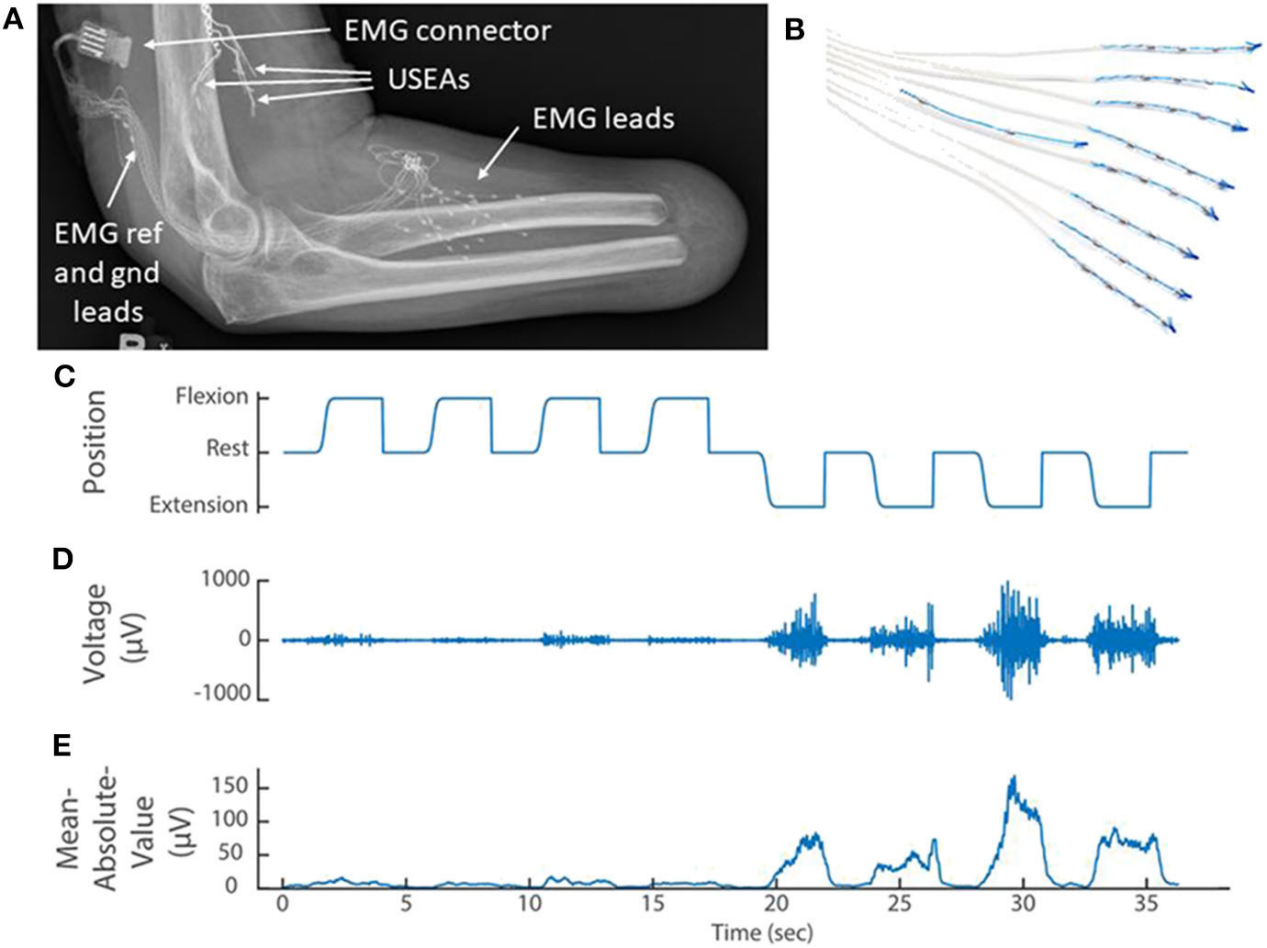
**(A)** X-ray of the elbow and residual forearm of a transradial amputee implanted with implanted three Utah Slanted Electrode Arrays (USEAs) and **(B)** 32 single-ended EMG leads (iEMG) and reference and ground. **(C)** The amputee mimics preprogrammed movements of the prosthesis (kinematics) while the portable Nomad system records iEMG voltage signals. **(D)** A representative iEMG channel that is active primarily during extension of the index finger. **(E)** A representative feature [mean absolute value of the iEMG channel in **(D)**] that is used to train the prosthetic control algorithm. Implanted Utah Slanted Electrode Arrays (USEAs) were not used with the portable system, but could be incorporated in future versions.

Training the prosthetic control algorithm [i.e., modified Kalman filter (George et al., 2019a)] first requires the user to mimic preprogrammed movements of the prosthesis as it cycles through several movement trials for each DOF (Figure 2C; George et al., 2020d). Features were then calculated for each differential EMG pair (496 total pairs from 32 single-ended electrodes, Figure 2D) by taking the mean-absolute value of a moving 300-ms window (Figure 2E). Using the kinematic positions and the EMG features, the portable computer chose 48 optimal features using the Gram-Schmidt forward-selection algorithm (Efron et al., 2004; Hwang et al., 2014; Nieveen et al., 2017) and computed the Kalman filter matrices (Wu et al., 2006). Forty-eight channels were used because, anecdotally, that number had consistently provided good control for in-lab experiments using our desktop system. Nieveen et al. suggests that a small improvement in performance could be achieved with a few more channels (e.g., 55), although this number will vary by participant and training session (Nieveen et al., 2017). Increasing the number of channels will also increase the control algorithm training and prediction times.

The Kalman filter presented by Wu et al. (2006) was modified to improve stability and reduce the effort required to sustain grasping movements by using an *ad-hoc* latching filter (Nieveen et al., in review). External, *ad-hoc* thresholds were also then applied as follows and as previously described in George et al. (2020a):

(1) $${\hat{\text{x}}}_{mod}=\{\begin{array}{ll} \frac{{\hat{\text{x}}}_{\text{new}}\cdot G-T}{1-T} & when\;{\hat{\text{x}}}_{\text{new}}\geq T\\ 0 & when\;{\hat{\text{x}}}_{\text{new}}<T \end{array}$$

where ${\hat{x}}_{new}$ is the output from the Kalman filter (defined to exist between −1 and +1), ${\hat{x}}_{mod}$ is the output with the modifications applied, G is the gain (set to 1), and T is the threshold (set to 0.2 for all DOFs). This equation is for the positive direction of each DOF (e.g., flexion, abduction, pronation); a similar equation that preserves the sign and directionality for the negative direction was applied accordingly. Note that the non-modified output (${\hat{x}}_{new}$) is fed recursively back to the Kalman filter to preserve stability while the modified output is only used to control prosthetic arm. In equation (1), ${\hat{x}}_{mod}$ is normalized to 0 to +1 (or −1, if in the negative direction) using the ′1 − *T*′ divisor.

### Human Subjects

In this manuscript, one amputee and two intact participants used the portable system. All participants used the system in the lab, but only the amputee and one of the intact participants used the system, under supervision, at home.

#### Transradial Amputee

For the amputee, eight iEMG leads (Ripple Neuro; Salt Lake City, Utah, USA) with four electrodes each, and a ninth lead with an electrical reference and ground, were implanted in lower-arm extensor and flexor muscles as described previously (George et al., 2019a; Figures 2A,B). The electrode connector exited through a percutaneous incision and mated with an active gator connector (Figure 2A; Ripple Neuro; Salt Lake City, Utah, USA). This participant also had Utah Slanted Electrode Arrays implanted in the median and ulnar nerves but these devices were not used with the portable system. Surgical details have been previously described (Wendelken et al., 2017; George et al., 2018, 2019a; Page et al., 2018).

#### Intact Participants

Intact individuals were able to use the portable system with a 3D printed, custom-made bypass socket (Paskett et al., 2019) and a custom-made neoprene sleeve with 32 sEMG electrodes, plus one reference and one ground (George et al., 2020b). Inexpensive, stainless steel-coated, marine grade, brass snaps were crimped into the neoprene to serve as dry electrodes and soldered to flexible wire for easy connection via a SAMTEC connector. The electrodes were roughly evenly spaced over flexor and extensor forearm muscles, about half on the flexors, and half on the extensors (covering about 8 inches distal to the elbow). Precise placement was not a concern as we relied on the Gram–Schmidt forward selection algorithm to choose the 48 most informative bipolar pairs for the motor decode algorithm (see section EMG feature calculation and decoding of motor intent).

As described previously (Paskett et al., 2019), the bypass socket is an open source device which suspends a prosthetic arm beneath the intact arm of the healthy volunteer and provides adequate range-of-motion so that the healthy volunteer can perform activities of daily living with an upper-limb prosthesis. The bypass socket was designed so that the electrode sleeve could be pulled up onto the forearm, locating the 32 recording electrodes over the extrinsic flexor and extensor hand muscles and the reference and ground electrodes over the ulna, about 2 cm distal to the elbow.

All experiments and procedures were performed with approval from the University of Utah Institutional Review Board.

## Results

### EMG Recordings Are Consistent Across Desktop and Portable Systems

To ensure that the EMG was stored correctly on the Nomad, we concurrently recorded EMG with the portable system and a laboratory desktop system in one intact participant while the participant completed a training session (394 s in length). The correlation coefficient was calculated after concatenating the sEMG data from all 32 recorded channels. As expected, concurrent recordings of sEMG on the portable and desktop systems were highly correlated (ρ = 0.95; *p* < 0.001) and the sEMG features (mean-absolute value sEMG data with a 300-ms window) were nearly identical between the two systems (ρ = 0.99; *p* < 0.001). Due to slight variation in clock speeds, a temporal delay was observed (about 100 ps/sample) which reduced the correlation coefficient. However, the correlation of the sEMG features suggests functional equivalency between the two recording systems.

### Portable System Offers a Simple User Interface and Customizable Control Options

**Figure 3:**
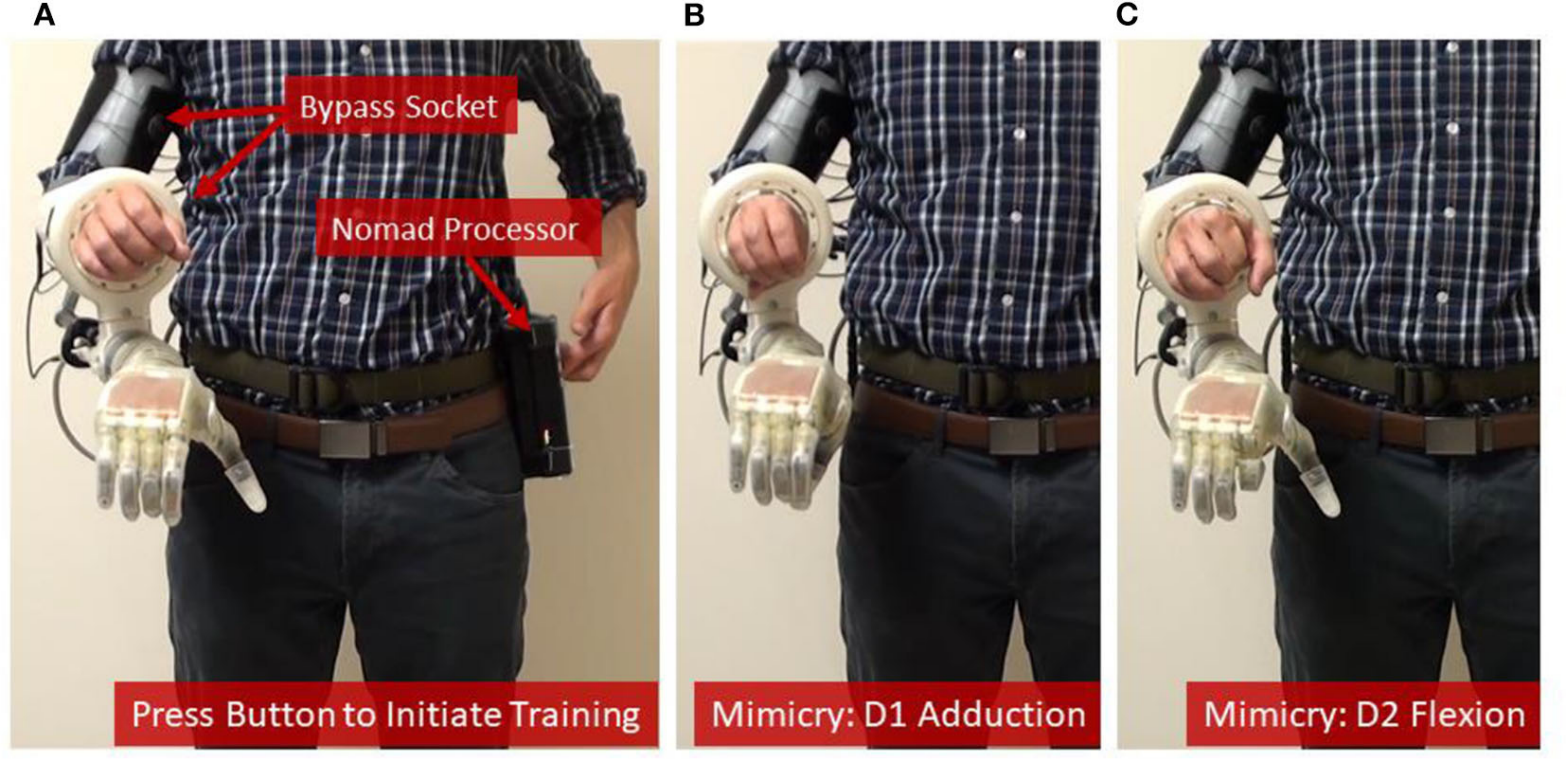
Training the prosthetic control algorithm with the portable system. **(A)** The user [shown here as an intact subject using a bypass socket (Paskett et al., 2019) to support the LUKE Arm] presses a button on the Nomad to start the training sequence, and then mimics the prosthesis while the Nomad cycles through each of six DOFs—**(B,C)** show D1 adduction and D2 flexion, respectively.

Three external buttons were employed to create a simple user-friendly interface. Pressing the first button initiated a new training session, which automatically granted control of the prosthesis to the user once training was complete (Figure 3A, see also Supplementary Video 1). The second button initiated a previously trained and compiled control algorithm (if available), so that the user could have on-demand control of the prosthesis. Finally, sequential inputs on a third button was used to toggle between position or velocity control modes or to freeze a DOF at a desired position.

### Portable System Can Be Trained Rapidly Using Steady-State Modified Kalman Filter

The system was trained in 7.5 min—including the time needed to collect the training data (4.2 min) and the subsequent channel selection and computation of the modified Kalman filter matrices (about 3.3 min) (Table 1). Timing data for Table 1 were recorded during training and testing with one intact participant in the lab. Because training data and the corresponding Kalman filter matrices have the same dimensions regardless of the user, the times listed in Table 1 are universal. Loop speeds were calculated as the average over a 16.5 second window (500 samples) while the user actively controlled the prosthesis. Training data included four trials of flexion and extension for D1, D2, D3/D4/D5, and the wrist; D1 adduction and abduction; wrist pronation and supination; and grasping and extending all digits together (Figures 3B,C, see also Supplementary Video 1). The trained modified Kalman filter was automatically saved to a log file and could be recompiled onto the Nomad as a stand-alone application for on-demand use (e.g., the second external button). This was accomplished over the Nomad's wireless network using a laptop and required <30 s.

**Table 1:** Computational times required for training and testing (running) the steady-state, modified Kalman filter on the portable system.

**Process**	**Computation time**
**Training**	
Data collection	252 s
Channel selection	198 s
Train steady state Kalman filter	0.7 s
Total Time	7.5 min
**Testing**	
Update positions	0.7 ms

Prior to use, the steady-state modified Kalman gain matrix (K) was calculated by iteratively running the filter until the fluctuations in each value of the gain matrix were <1 × 10^−6^, reaching steady state after about 25 ms. With the gain (K), the observation (*H*) and the state-transition (*A*) matrices, a steady state matrix (Γ) was then calculated:

(2) $$\Gamma =A-K^{*}H^{*}A$$

Thus, new position predictions (${\hat{x}}_{new}$) were calculated with only two matrix multiplications involving the previous positions (${\hat{x}}_{previous}$) and 48 EMG features (*z*):

(3) $${\hat{x}}_{new}=\;\Gamma^{*}{\hat{x}}_{previous}+K^{*}z$$

This simplification avoided a computationally expensive matrix inversion required by the recursive algorithm. Consequently, the time required to predict new positions and update the prosthesis was on average <1 ms, far below the update loop speed of 33 ms (Table 1). If the user desires, a velocity control mode for any DOF can also be provided using the position output from the Kalman filter (${\hat{x}}_{new}$):

(4) $${\hat{x}}_{velocity}=\;{\hat{x}}_{velocity}+{\hat{x}}_{new}{\;}^{*}\Delta t^{*}\gamma$$

where Δ**t** is the loop speed (33 ms) and γ a dampening factor (set to 0.95). In our previous experiments, some amputee subjects have preferred specific DOFs, such as wrist rotation or wrist flexion, to operate in velocity mode (Kluger, 2019).

### Portable System Can Be Used at Home to Complete Various Activities of Daily Living

**Figure 4:**
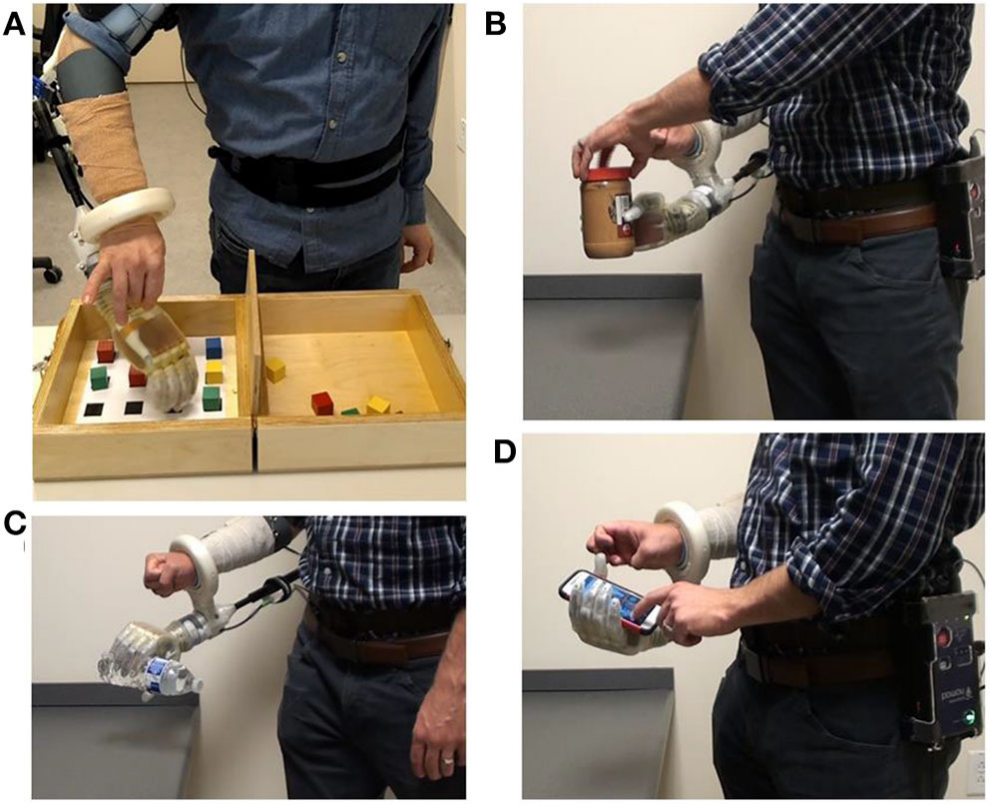
After the motor control algorithm was trained, intact participants used the portable system with a bypass socket in the lab to perform **(A)** an arm dexterity test and activities of daily living: **(B)** opening a jar; **(C)** pouring motion; and **(D)** using a smart phone.

**Figure 5:**
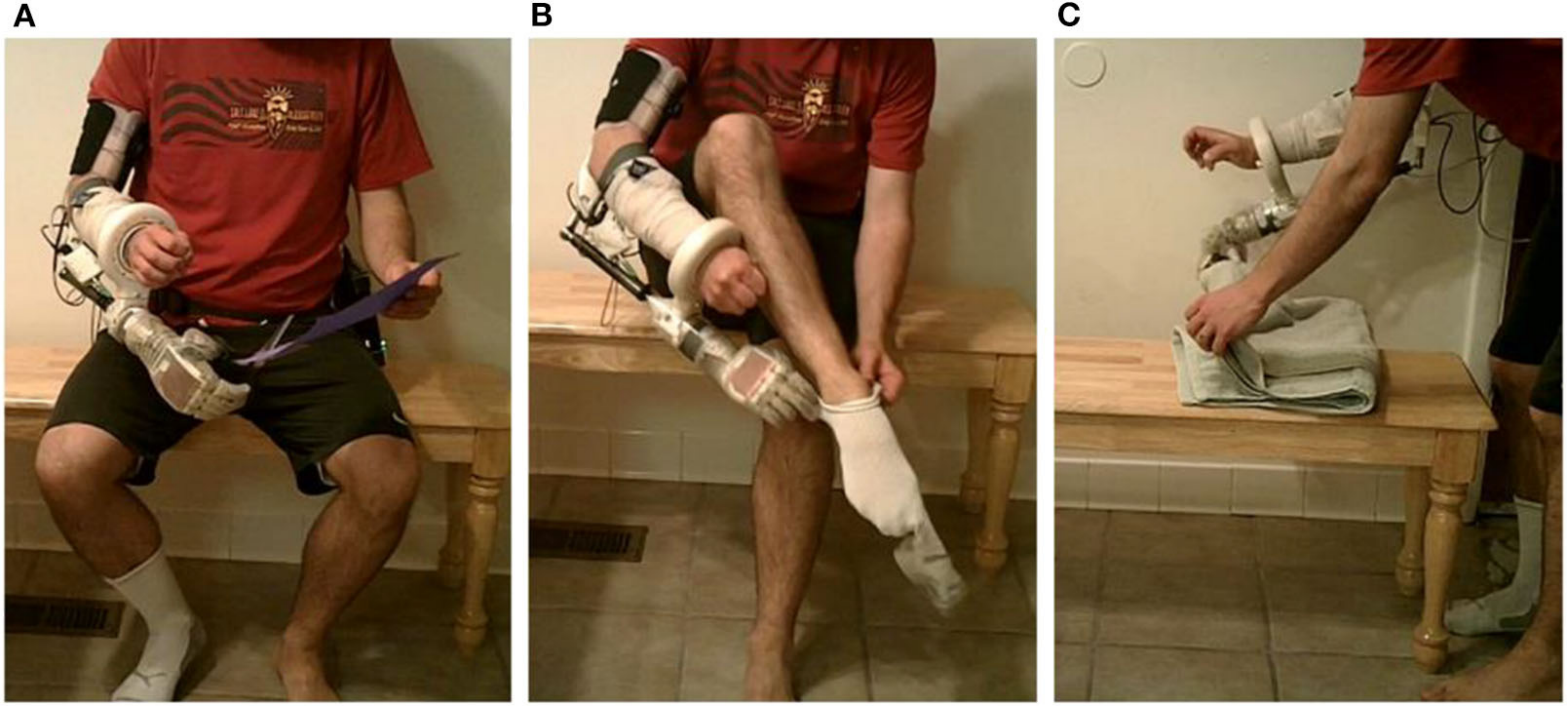
Two-handed activities of daily living at home using a bypass socket and the portable system: **(A)** using scissors; **(B)** donning a sock; and **(C)** folding a towel.

**Figure 6:**
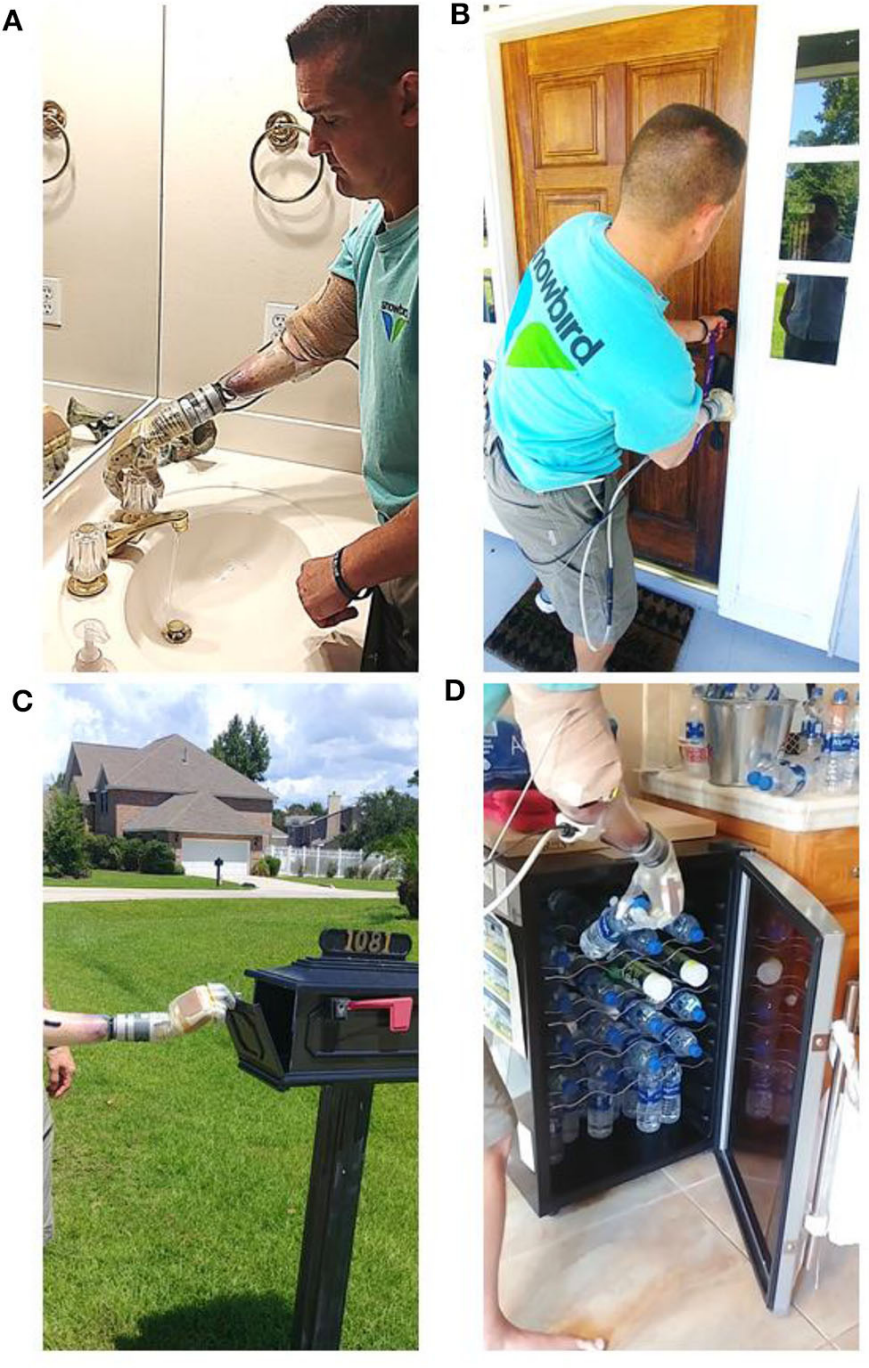
Transradial amputee performed supervised activities of daily living, of his own choice, at home using the portable take-home system. Images show the participant **(A)** turning faucet in the bathroom; **(B)** locking the dead-bolt on the front door; a bi-manual task not possible with his commercial prosthesis; **(C)** opening the mail box; and **(D)** retrieving water from the refrigerator.

The portable system was used by both intact participants to perform arm dexterity tests and activities of daily living in the lab (Figure 4), as well as by one intact participant to perform two-handed tasks at home (Figure 5). One transradial amputee used the system at home, under staff supervision, to perform tasks of his choosing, some of which were not possible with his commercial prosthesis (Table 2 and Figure 6). Table 2 shows that the most common movements used were grasp (D1–D5 flexion) and pinch (D1 and D2 flexion) in combination with the wrist movements. Several successfully completed tasks were not listed in Table 2 because of similarity to other tasks (e.g., picking up another dog toy, pill bottle, TV remote, or a potato from the pantry; or turning on exterior faucet).

**Table 2:** List of tasks chosen by the amputee to attempt at home using the portable system.

**Task**	**Completion time**	**Detailed description**
**Successful**		
Lock front door (dead-bolt)^†^	13 s	Used grasp (D1–D5 flexion) to grab and pull the door toward him so the bolt lined up and could be locked using intact arm
Open front door	7 s	Used grasp to pull down on the handle and to pull door open
Open refrigerator and retrieve water bottle	25 s	Used grasp and wrist flexion/extension to open refrigerator, grasp water bottle, and transfer to intact arm
Open oven door	16 s	Used grasp and wrist flexion/extension to grab the handle, open, and then shut the door
Turn on bathroom faucet	29 s	Used both grasp and pinch (D1 and D2 flexion) while turning faucet with gross arm movement
Open cabinet doors	22 s	Used precise pinch and wrist flexion/extension to grab small handles and pull doors open (2 doors)
Pick up dog toy	8 s	Used grasp and wrist flexion/extension to pick up a dog toy, and hold it for dog to bite
Put on shoe^†^	22 s	Used grasp and wrist flexion to hold shoe tongue while donning shoe held with the intact arm. Included a release and readjustment of the grip on the shoe tongue
Move garbage can	9 s	Used grasp and wrist flexion/extension to grasp the garbage can handle and move it about 10 feet
Check for mail at box	8 s	Used pinch and wrist extend to open mail box (as if he were to put/take mail) and then close with intact arm
**Failed**		
Input garage code on key pad	NA	This task required a pointed finger position (D2 extended; D1, D3, D4, and D5 flexed) which, was not included in the training. The amputee could make the motion; however, it was not stable enough to successfully complete task
Turn on push button oven light	NA	This task required a pointed finger position (D2 extended; D1, D3, D4, and D5 flexed) which was not included in the training. Amputee could make the motion; however, it was not stable enough to successfully complete task

† *Denotes a task where amputee used the prosthesis and their intact hand simultaneously*.

### Rich Dataset From Portable System Reveals Novel Information About Prosthesis Use

**Figure 7:**
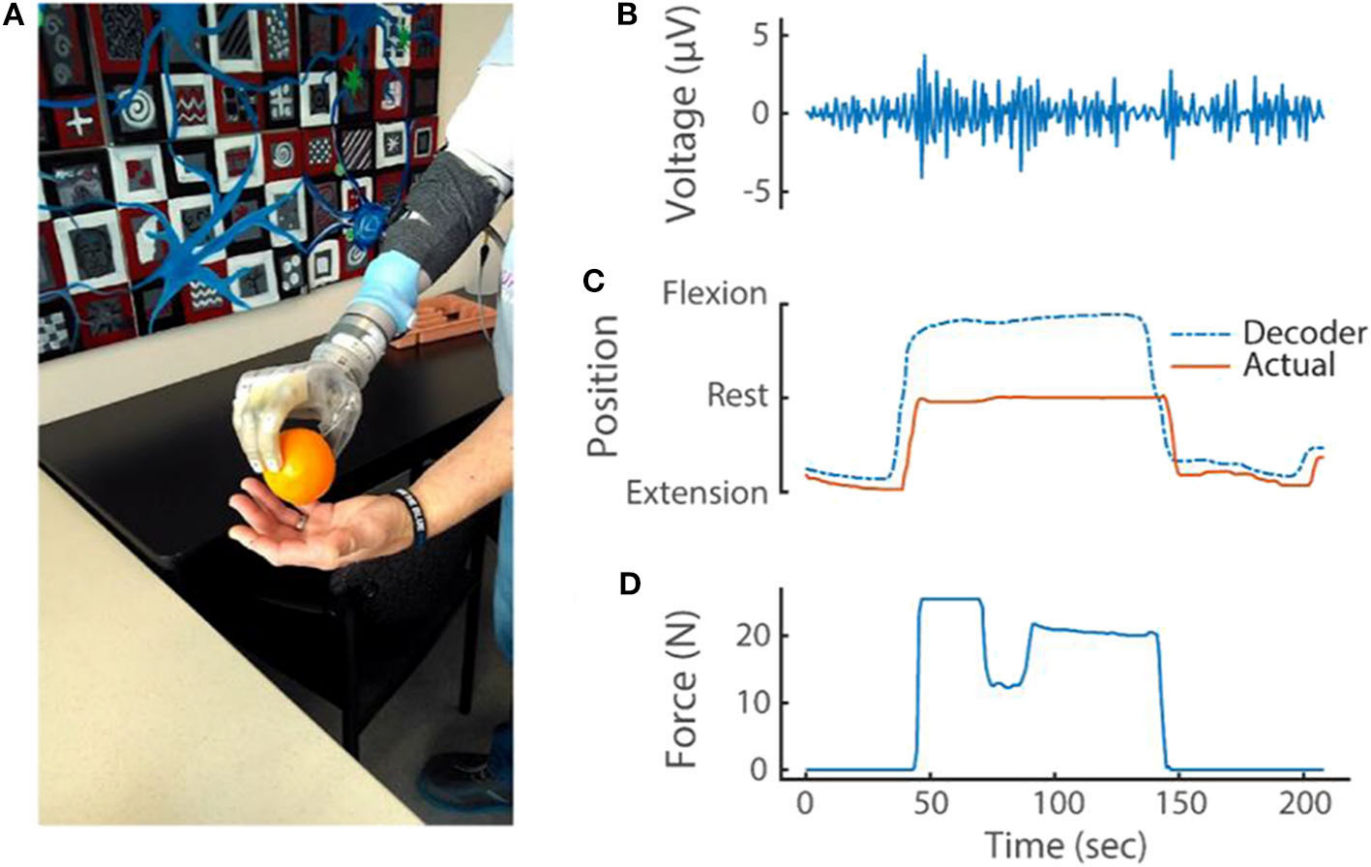
**(A)** Transradial amputee picking up an orange using implanted EMG electrodes and the portable system. During this task, the portable system recorded and stored **(B)** differential EMG (at 1 kHz), **(C)** kinematic output of the modified Kalman filter and actual kinematics of the prosthesis (at 30 Hz) and **(D)** prosthesis sensor values (at 30 Hz). For simplicity, only one differential EMG channel (of 48 total) and only one sensor (D1 pressure sensor; out of 13 total pressure and torque sensors and six DOFs) are shown.

EMG (sampled at 1 kHz), kinematic positions and forces applied to the prosthesis (both sampled at 30 Hz) were stored on the Nomad while a transradial amputee grasped, held and released an orange (Figure 7; see also Supplementary Video 4). Three phases of movement were clearly identified: preparing to grasp (when the index finger is near full extension); grasping (where the algorithm predicted the finger to be near full flexion but the orange restricted the actual position to about the rest position, which resulted in a dramatic increase in force); and releasing the orange (where the finger extended toward near full extension).

Data are saved at a rate of 250 MB/h in an ‘.hd5’ format. As a result, the 500 GB capacity of the Nomad can record nearly 2,000 h of arm use.

## Discussion

We have described a portable, prosthetic control system and the first at-home use of a multi-degree-of-freedom, proportionally controlled bionic arm. The system uses a modified Kalman filter to provide real-time, proportional control—including independent, and simultaneous movement—across 6 DOFs. We have shown that the modified Kalman filter can be trained in 7.5 min using the Nomad, a portable electrophysiological recording system equipped with an ordinary processor. In addition, the time needed to acquire EMG and compute and update the prosthetic arm positions was <1 ms on average—far below the 33 ms update cycle—and provided real-time movement updates for the users.

The portable system also stores EMG, position and force data with unprecedented temporal resolution. This comprehensive dataset will be crucial for fully understanding how proportional control algorithms are used during unsupervised at-home use. Because of the Nomad's large storage capacity and USB and Bluetooth connections it could also be configured to collect and store other types of data (e.g., video, bilateral arm use with IMUs).

To study at-home prosthetic use, previous take-home systems have stored limited usage data, including the time the device was turned on (Graczyk et al., 2018; Simon et al., 2019), aggregated hand movement (Simon et al., 2019), how often specific predefined grasps were used (Kuiken et al., 2016; Hargrove et al., 2017; Simon et al., 2019) or force applied to a limited number of sensors on the hand (Graczyk et al., 2018). Although these approaches may be sufficient for less refined control algorithms, to fully understand how proportional control is used, both high-temporal-resolution kinematic and force data for each DOF are necessary.

The example in Figure 7 highlights how the comprehensive data recorded reveals complex interactions between the various DOFs with proportional control. The stable D2 kinematics implies that the amputee held the orange with a fixed grasp from pick up to release; however, the force data revealed a dip in force during this same period. Close inspection of the kinematics from the opposing D1 also shows that a subtle readjustment occurred to improve the grasp stability (this can be seen in Supplementary Video 4). These refined movements are possible because of proportional control algorithms. Because DOFs are coupled together during object manipulation, the connection between each DOF must be considered.

Rich datasets like this will help researchers and clinicians study at-home, unsupervised use; improve prosthetic control algorithms, and training paradigms (George et al., 2018) by understanding the types of grasps and DOFs commonly used; understand when mastery of prosthesis control occurs and when interventions might be applied or lifted; better describe noise encountered in real-world environments and design features and algorithms that reduce its influence on motor performance; and address many other unanswered questions about at-home use of advanced upper-limb prostheses. These rich datasets will also enable future at-home trials to study the benefits and use of high-resolution sensory feedback from intraneural electrical stimulation—a feature soon to be added to the portable system.

In contrast, previous data collection during at-home prosthetic use has relied on subjective surveys, usage logs, IMUs, and the amount of time the device is turned on to describe prosthetic use (Hargrove et al., 2017; Resnik et al., 2017, 2018b; Graczyk et al., 2018). However, these approaches only approximate actual prosthesis use and could be misinterpreted. Some pattern recognition studies have recorded kinematic output and use of predefined grasps (Kuiken et al., 2016; Simon et al., 2019).

Two of the tasks successfully completed by the amputee at home required use of the intact arm along with the prosthesis—donning a shoe and locking the front door (Table 2). However, other tasks on the list could also be two-handed, such as using the intact hand to remove mail from the box or food from the refrigerator or oven while holding the door open with the prosthesis. The two tasks where the amputee was unsuccessful required a pointed finger position (D2 extended while D1, D3, D4, and D5 were flexed). Even though this combination was not included in the training sequence the amputee was able to position the digits appropriately; however, the positions were not stable enough to complete the task. Both of these tasks also required the amputee to lift and hold the heavy prosthesis vertically, which could have also added instability to the control.

The most common arm movements used in the at-home setting where opening and closing the hand, or pinching the thumb and index finger, in combination with wrist movements. Combination movements can be performed simultaneously because the Kalman filter algorithm assumes independent DOFs. To simplify control, the participant controlled the wrist with a velocity mode (while the digits were in position control)—allowing the user to first set a wrist position prior to completing the grasp or pinch, if desired.

Because combination movements involving wrist were so prevalent during at-home use, we recently studied the benefits of training the modified Kalman filter with combination movements involving the wrist, in addition to single DOF movements (Paskett et al., in review). We found that combination training sequences provide the user with improved, intuitive wrist position control during simultaneous movements. As a result, our future studies will also include wrist combination movements.

An important aspect of the portable system is the fast computation of position updates using a steady-state Kalman filter. We initially implemented the full recursive Kalman filter within each update cycle. However, the time required to complete the update was near and often exceeded our 33-ms update loop speed. Updating movement positions with the steady-state Kalman filter was quick (less than our loop time) and straightforward to implement. Malik et al. performed a rigorous comparison of the steady-state and full recursive Kalman filter using neural spike data and concluded that after steady-state convergence the two predictions are essentially identical (Malik et al., 2011). Our fast position update speeds will allow additional features to be added, including high-resolution, biomimetic, sensory feedback from intraneural (Wendelken et al., 2017; George et al., 2019b) or electrocutaneous (George et al., 2020a) stimulation.

The most computationally demanding aspect of training was performing Gram-Schmidt forward selection to choose the 48 most useful features out of the 496 differential pairs. Despite taking considerable time up front, this down-selection method has several advantages (Nieveen et al., 2017). First, choosing the features up-front enables fast loop speeds (below 33 ms) by eliminating the need to calculate complex features (e.g., principal components) or even all 496 differential EMG features during each update cycle. Second, forward selection recursively selects features that are maximally correlated with the training kinematics and minimally correlated with each other by orthogonalizing the remaining channels after each channel is selected. This ensures that each selected feature describes kinematics and not uncorrelated noise. Refined movements, the hallmark of proportional control algorithms, account for little variance and could be inadvertently discarded using techniques agnostic to the training kinematics. Finally, orthogonalization in the forward selection algorithm avoids redundant features and singularities.

It was possible to avoid the need for down-selection and only use the original 32 single-ended features. However, by calculating all possible differential pairs, signal from a specific muscle might be better isolated from unwanted signal or noise and identified by the forward selection algorithm. Indeed, when we allow the forward selection algorithm to choose from among both the 32 monopolar and 496 bipolar pairs—which is the case in our lab desktop system but not the portable system—the monopolar channels are rarely selected.

Importantly, within 8 min of powering the system on, the user can have real-time proportional control of six DOFs. The amount of time required to both collect training data by mimicking preprogrammed movements and to train the prosthetic control algorithm are related to the number of trials for each mimicked movement. In this work, and published elsewhere (George et al., 2019a), an amputee familiar with the training process trained with only four trials on each DOF and a grasp and extension of all digits. With this training, he was able to control the prosthesis in the lab and perform tasks not possible with his commercial prosthesis at home (George et al., 2019a). A less experienced user may require training with more trials; however, even if a naïve user requires twice as many trials, the total training time is still under 15 min.

An important question we have yet to fully explore is how often will retraining be required? The amputee in this study successfully used a trained algorithm the following day, suggesting daily training may not be necessary. It is also reasonable to believe that training with data collected across multiple days could provide better control (George et al., 2020b), especially considering that iEMG leads are relatively stationary when implanted into residual musculature. However, training daily provides new users with the best control algorithm given the EMG features collected on that day and our experience suggests that over time they will become more stereotyped and thus have improved control. From the start, our training requires less time than pattern recognition algorithms which can require 14–40 h of upfront, in-lab training with experienced professionals (Resnik et al., 2017, 2018a).

Currently, to use a previously trained control algorithm, the modified Kalman filter's parameters must be recompiled into a stand-alone application on the Nomad. Although this process is very fast (<30 sec) and wireless, it currently requires an external computer running MATLAB^®^. Planned future work includes the ability to directly load trained parameters from a local file stored on the Nomad for on-demand use.

Ultimately, the ability to communicate with an application running on the Nomad was limited to one button (the other two could only start and stop a compiled application). Thus, we implemented sequential button pressing to selectively lock an individual DOF. The amputee used this feature to lock wrist rotation when using the system at home. For this amputee, poor wrist control was not uncommon and was likely due to dystonic muscle activity, common among those afflicted with complex regional pain syndrome and multi-year arm disuse prior to amputation (George et al., 2018). However, despite the amputee's having low-amplitude EMG signals (e.g., Figure 7B), the modified Kalman filter algorithm provided control for five degrees of freedoms. Intact users did not lock any DOFs and had all six, independent, proportionally controlled DOFs.

Near the end of Supplementary Video 4, the amputee displays difficulty releasing the orange. Release is normally an easy task, requiring only muscle relaxation for the position control algorithm to move the digits into an open resting position. However, delayed object release in this case was due to the participant's dystonia—he could not relax muscle easily for the hand to open. This did not occur often, but dystonia was more prevalent during some lab visits than others.

Future improvements will include wireless communication to a tablet or phone app where control selections can be easily made, communicated and saved locally on the Nomad. This will enable real-time adjustments including setting specific DOFs to velocity mode; adjusting the *ad-hoc* gains and thresholds of the modified Kalman filter on a DOF-by-DOF basis; and reloading a previous training or retraining the prosthesis with a modified training protocol if the first training was not satisfactory.

In its current form, the portable system is programmed to communicate only with the DEKA LUKE Arm. However, other custom communication sockets could be designed to communicate through the micro D-sub, USB or Bluetooth connections available to Nomad for proportional control of and data logging from other prosthetic limbs.

The current system also has significant cabling that connect the DEKA LUKE Arm to its battery and the Nomad to the DEKA LUKE Arm and the Ripple front-end amplifier. Upcoming, at-home, unsupervised studies will likely require a supportive partner/care-taker to assist the amputee when donning the equipment and to aid in securing the cables. The take-home study will begin with an acclimation phase where the amputee (and partner/care-taker) receive in-lab and at-home supervised training prior to unsupervised use. We also envision a hip or back-pack where the Nomad, batteries and excess cable length can be organized and housed. In the future, wireless communication between the Nomad, implanted electrodes and prosthesis could eliminate cables and provide amputees with greater independence.

The importance of reliability in a take-home system cannot be understated—software and hardware must function as intended in the everyday environment. To fully test reliability, the system must be used at home, over many days and for many uses. To date, the system has been used on numerous occasions on campus and in the lab, but only at home by our laboratory staff and by one transradial amputee under staff observation (George et al., 2019a). Ultimately, this system will be used in upcoming take-home clinical trials to record high-resolution data and study advanced, proportional control algorithms for upper-limb prosthesis use.

## Data Availability Statement

All datasets presented in this study are included in the article/Supplementary Material.

## Ethics Statement

The studies involving human participants were reviewed and approved by University of Utah Institutional Review Board. The patients/participants provided their written informed consent to participate in this study. Written informed consent was obtained from the individual(s) for the publication of any potentially identifiable images or data included in this article.

## Author Contributions

MB, EB, and TD designed the software running on the portable system. MB, TD, MP, and JG tested the system with the human participants. MB wrote the manuscript. All authors revised the manuscript. GC oversaw all aspects of this research.

## Conflict of Interest

EB was an employee of Ripple Neuro, during the development of the portable system. The remaining authors declare that the research was conducted in the absence of any commercial or financial relationships that could be construed as a potential conflict of interest.
